# The complete chloroplast genome of *Scutellaria indica* var. *coccinea* (Lamiaceae), an endemic taxon in Korea

**DOI:** 10.1080/23802359.2019.1640649

**Published:** 2019-07-13

**Authors:** Yoonkyung Lee, Sangtae Kim

**Affiliations:** Department of Biology, Sungshin University, Seoul, Korea

**Keywords:** Chloroplast genome, genome skimming, endemic species, Korea, *Scutellaria indica* var. *coccinea*

## Abstract

*Scutellaria indica* var. *coccinea* (Lamiaceae) is an endemic taxon in Jeju Island, Korea. We present the complete chloroplast genome sequence of *S. indica* var. *coccinea*. Its genome size is 151,956 bp in length comprising a large single copy of 83,951 bp, a small single copy of 17,539 bp, and a pair of inverted repeats measuring 25,233 bp. The complete genome contains 115 genes including 80 protein-coding genes, four rRNAs, and 31 tRNAs. Phylogenetic analysis of this species with 10 published chloroplast genomes in Lamiaceae showed that *S. insignis* is a sister to *S. indica* var. *coccinea*.

*Scutellaria* L. is the second largest genus in the Lamiaceae with approximately 470 recognized species that are mainly distributed in temperate regions. Plants are annual or perennial herbs and rarely subshurubs or aquatic species. Eighteen taxa of *Scutellaria* are distributed in Korea (Kim and Lee 1995; Yang 2004; Kim et al. 2011). Three of them are endemic species in Korea: *S. indica* var. *coccinea* S. Kim & S. Lee, *S. insignis* Nakai, and *S. pekinensis* var. *maxima* S. T. Kim & S. Lee. Especially, *S. indica* var. *coccinea* is found only in a small volcanic crater (Sangumburi) of Jeju Island, which is located to the south of the Korean peninsula (Kim and Lee 1995).

The recent classification system of *Scutellaria* includes subgen. *Scutellaria* and subgen. *Apelthanthus* containing seven sections (Paton 1990). In this system, 34 unranked morphological ‘species-groups’ have also been suggested under *Scutellaria,* which is the largest section in the genus (ca. 240 species). In this species-group category, *S. indica* L. is included under ‘*S. violacea* species-group’ of the section *Scutellaria,* which is characterized by nutlets with acuminate papillae terminating in hooks. In *S. indica*, *S. indica* var. *coccinea* is distinguished from other varieties having widely cordate leaves, conspicuously sunken veins in adaxial leaf surface and dark pink corolla color.

In this study, we report a complete sequence of cp genome of *S. indica* var. *coccinea* (GenBank accession: MN047312). Genomic DNA was extracted from leaves of a plant cultivated in Sungshin University, which is propagated from seeds of a wild plant collected in Jeju Island (N33°25′57.33″, E126°41′26.72″). Voucher specimen is deposited in Sungshin University herbarium (*S. Kim 2015-0298*). The whole genome sequencing was conducted with 100 bp paired-end reads on the BGISEQ-500 sequencer (BGI, Shenzhen, China). To obtain the chloroplast genome (cp) sequence, paired-end reads were mapped against a reference cp genome (GenBank accession: NC_028533) which is previously reported from *Scutellaria insignis* using Geneious (v9.0.5; Kearse et al. 2012). The quality of a consensus sequence and its mapping condition were examined visually. The cp genome was annotated using GeSeq (Tillich et al. 2017).

The complete cp genome of *S. indica* var. *coccinea* is 151,956 bp in length, containing a large single copy (LSC) of 83,951 bp, a small single copy (SSC) of 17,539 bp, and a pair of inverted repeats (IR) measuring 25,233 bp. It includes 115 genes comprising 80 protein coding genes, four rRNA genes and 31 tRNA genes. Among these genes, 16 genes carry a single intron, and two genes (*clpP* and *ycf3*) contain two introns, respectively.

The phylogenetic tree was reconstructed with this cp genome and previously published cp genomes (three in *Scutellaria* and seven in other Lamiaceae; Figure 1). The maximum-likelihood analysis is performed using raxmlGUI (v.1.5; Silvestro and Michalak 2012). In the phylogenetic tree (Figure 1), *S. indica* var. *coccinea* is a sister to *S. insignis*. In this study, we report the cp genome of *S. indica* var. *coccinea* as an endemic taxon in Korea. The findings contribute to the conservation of this species and the phylogenetic study of *Scutellaria*.

**Figure 1.**
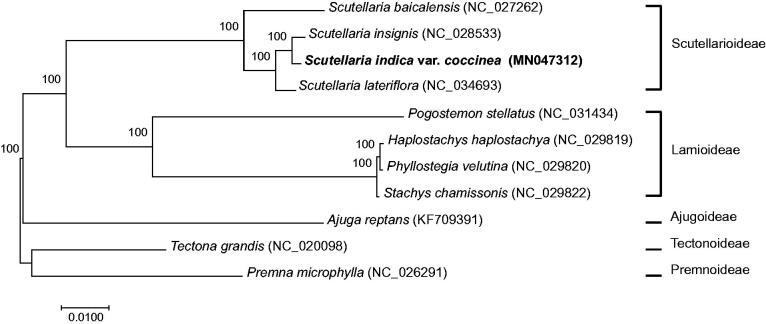
A maximum-likelihood tree based on ‘GTR + gamma + I’ model using the cp genome of *S. indica* var. *coccinea* and 10 selected cp genomes reported in Lamiaceae. Numbers above the node indicate bootstrap values.
